# Predicting Factors of Plasma HIV RNA Undetectability after Switching to Co-Formulated Bictegravir, Emtricitabine, and Tenofovir Alafenamide in Experienced HIV-1 Patients: A Multicenter Study

**DOI:** 10.3390/v15081727

**Published:** 2023-08-12

**Authors:** Monica Basso, Giuliana Battagin, Stefano Nicolè, Maria Cristina Rossi, Francesco Colombo, Nicole Pirola, Stefano Baratti, Silvia Storato, Federico Giovagnorio, Vincenzo Malagnino, Grazia Alessio, Antonio Vinci, Massimo Maurici, Loredana Sarmati, Saverio Giuseppe Parisi

**Affiliations:** 1 Department of Molecular Medicine, University of Padova, Via Gabelli, 63, 35100 Padova, Italy; monica.basso@unipd.it (M.B.); francesco.colombo@studenti.unipd.it (F.C.); nicole.pirola@studenti.unipd.it (N.P.); federico.giovagnorio@studenti.unipd.it (F.G.); 2 Infectious Diseases Unit, Vicenza Hospital, 36100 Vicenza, Italy; giuliana.battagin@aulss8.veneto.it (G.B.); stefano.nicole@aulss8.veneto.it (S.N.); 3 Infectious Diseases Unit, Treviso Hospital, 31100 Treviso, Italy; mariacristina.rossi@aulss2.veneto.it; 4 Infectious Diseases Unit, Venezia Hospital, 30122 Venezia, Italy; stefano.baratti@aulss3.veneto.it (S.B.); silvia.storato@aulss3.veneto.it (S.S.); 5 Infectious Disease Unit, Department of System Medicine, Tor Vergata University and Hospital, 00133 Rome, Italy; vincenzo.malagnino@uniroma2.it (V.M.); grazia.alessio@students.uniroma2.eu (G.A.); srmldn00@uniroma2.it (L.S.); 6 Doctoral School in Nursing Science and Public Health, University of Rome “Tor Vergata”, 00133 Rome, Italy; antonio.vinci.at@hotmail.it; 7 Department of Biomedicine and Prevention, University of Rome “Tor Vergata”, 00133 Rome, Italy; maurici@med.uniroma2.it

**Keywords:** co-formulated bictegravir, emtricitabine and tenofovir alafenamide, experienced HIV-1-positive patients, HIV RNA undetectability, HIV RNA control prior to switch, CD4+ nadir, HBcAb positivity

## Abstract

Switching to bictegravir, emtricitabine, and tenofovir alafenamide (BIC/FTC/TAF) from other antiretroviral regimens is safe and effective for virologically suppressed people living with HIV (PLWH). The term virological suppression includes both low but detectable HIV viremia and undetectable HIV viremia, and the latter is possibly associated with a lower immune activation state. Herein, we describe a 24-month follow-up of experienced PLWH with plasma HIV RNA undetectable or detectable < 50 copies/ml switching to BIC/FTC/TAF. A previous 12-month monitoring was available, and the factors correlated with treatment efficacy. This retrospective multicenter study included PLWH who switched to BIC/FTC/TAF in the period of 2019–2022, and who were HBsAg and HCV RNA negative. The follow-up study times were 6 (T6), 12 (T12), 18 (T18), and 24 (T24) months after the switch (T0). Survival analysis with multiple-failure-per-subject design, Kaplan–Meier survival estimates, multivariate analysis of variance, multilevel linear regression, and a hierarchical ordered logistic model were applied. A total of 329 PLWH had plasma HIV RNA which was either undetectable or detectable at <50 copies/mL at T0, and 197 responded to all inclusion criteria: M/F 140/57; the median CD4+ cell count was 677 cells/mm^3^; and HIV RNA at T0 was undetectable in 108 patients. Most of the 197 patients (122, 61.9%) were on a previous INSTI-based regimen. HIV RNA undetectability was more frequent at each follow-up point in patients with HIV RNA that was undetectable at T0, and it showed a higher frequency throughout the follow-up period in patients with always-undetectable HIV RNA in the 12 months before the switch. A higher nadir CD4 cell count had a predictive role, and HBcAb positivity had no influence. In conclusion, the switch could be programmed and possibly delayed on a case-by-case basis in order to achieve persistent plasma HIV RNA undetectability. Undiagnosed loss of HBcAb has no detrimental consequences on the response to BIC/FTC/TAF.

## 1. Introduction

HIV infection has become a manageable chronic condition thanks to the availability of antiretroviral therapy (ART); almost all people living with HIV (PLWH) achieve durable and maximal suppression of HIV replication with proper therapy and adherence and, therefore, have a life expectancy approaching that of HIV-negative patients [1,2]. For this reason, currently, virologic response is not the only goal of ART; quality of life improvement, the need to reduce long-term toxicities, and newly diagnosed chronic comorbidities are all factors involved in the choice of drug regimen. PLWH on successful ART may have an indication to switch to another ART combination [3]. Co-formulated bictegravir, emtricitabine, and tenofovir alafenamide (BIC/FTC/TAF) is a single-tablet three-drug regimen proven to be potent and well-tolerated in randomized international trials, including in virologically suppressed HIV-positive adult patients on antiretroviral therapy who were switched to this regimen [4,5]. It has a low risk of renal toxicity and possible drug–drug interactions, and a pharmacokinetic enhancer is not required; furthermore, it is active against HBV coinfection [6].

Real-life studies on treatment-experienced patients switched to BIC/FTC/TAF have defined viral suppression as the presence of a plasma HIV-RNA value ranging from 50 copies/mL [7,8,9] to 200 copies/mL [10,11], a choice mirroring the threshold beyond which virological failure is defined by the European AIDS Clinical Society [12] and the International Antiviral Society–USA [13], respectively. Notably, this definition includes both patients with very low-level viremia [14,15] and PLWH with undetectable viremia in the same category of virological responders. Identifying patients with complete suppression of viral replication according to routine laboratory testing is an inexpensive approach to analyzing the roles of the factors known to influence responses in patients switching to another regimen for reasons other than virological failure, and to describing the immune response in PLWH with possible lower chronic immune activation and reduced reservoirs with respect to PLWH with very low-level viremia [16,17,18].

The purpose of this study was to describe the 24-month follow-up of HIV treatment-experienced patients with plasma HIV RNA that is undetectable or detectable at <50 copies/mL at the time of switching to BIC/FTC/TAF. It focuses on factors correlated with treatment efficacy in the two patient cohorts, including the influence of plasma HIV RNA control in the 12 months before starting BIC/FTC/TAF.

## 2. Materials and Methods

### 2.1. Study Design

This retrospective cohort study was conducted at 4 (Rome, Treviso, Vicenza, and Venice) Italian Infectious Diseases Units and enrolled outpatients who were on successful ART and who had been switched to the drug combination BIC/FTC/TAF in the period 2019–2022. The decision to start the new regimen was up to the treating physician according to the updated treatment guidelines [19,20,21,22] and was performed as a routine clinical practice. Inclusion criteria were plasma HIV RNA < 50 copies/mL at the time of viro-immunological testing performed less than one month before the switch and only one viral load before the switch was performed; negative HBsAg; and undetectable HCV RNA (because of never having been HCV infected, spontaneous clearance, or due to sustained virological response after antiviral treatment). Plasma HIV RNA performed less than one month before the switch corresponded to T0.

The study was approved by the Ethics Committee of Padova Province (protocol code 0033219, approved on 16 May 2019) and was performed in accordance with Good Clinical Practice and the Declaration of Helsinki. The patients gave written informed consent.

### 2.2. Variables and Assessment Methods

Baseline demographic and clinical characteristics were collected, including age, gender, ethnicity, age at diagnosis, age at switch, hepatitis B core antibody, hepatitis C serology, absolute number of CD4+ T cell count at nadir, CD4+ T cell count at switch, and ongoing regimen before switch. The time of the switch was defined as the study’s T0 point.

Only patients with plasma HIV RNA < 50 copies/mL at the testing before the switch were included in the study. They were categorized as patients with undetectable plasma HIV RNA and as patients with plasma HIV RNA detectable but <50 copies/mL.

The control of plasma HIV viremia in the 12 months before the switch was categorized into the following groups: not detected (undetectable plasma HIV viremia in all the tests); plasma HIV viremia < 50 copies/mL (at least one test with a value < 50 copies/mL); and plasma HIV viremia ≥ 50 copies/mL (at least one test with a value ≥ 50 copies/mL).

For this study, the reasons for the switch were classified as follows: (1) treatment optimization; (2) simplification to a single-tablet regimen; (3) drug unavailability; (4) side effects; (5) drug–drug interactions, (6) prevention of cardiovascular risk; (7) risk of virologic failure—blip defined as a single plasma HIV RNA value between 50 and 500 copies/mL followed by an undetectable viral load [23] or residual HIV viremia, defined as plasma HIV RNA values ranging from 50 to 100 copies/mL [24]; (8) patient preference; and (9) unknown (not described). More than one reason for treatment change could be reported. The follow-up study times were 6 (T6), 12 (T12), 18 (T18), and 24 (T24) months after the switch to BIC/FTC/TAF, and plasma HIV viremia and CD4+ cell count were evaluated at each follow-up study time. Plasma HIV RNA was classified as undetectable, detectable at <50 copies/mL, detectable ranging from 50 to 100 copies/mL, detectable ranging from 101 to 1000 copies/mL, or detectable > 1000 copies/mL.

### 2.3. Statistical Analysis

Conventional descriptive analysis was employed by reporting raw values, alongside mean and median values for quantitative data and percentages for qualitative data. A multifaceted approach was used for inferential analysis, depending on the outcome of interest. Three cohorts of patients were identified on the basis of plasma HIV RNA control in the 12 months before the switch: always undetectable HIV RNA; plasma HIV viremia < 50 copies/mL; and plasma HIV viremia ≥ 50 copies/mL. The threshold of significance was set at *p* < 0.05 for inferential analysis.

The treatment efficacy, in terms of maintaining undetectable or low viremia after treatment, was reported as survival analysis with multiple-failure-per-subject design. Kaplan–Meier survival estimates were applied to analyze the response to treatment at the 4 follow-up study times (T6, T12, T18, and T24). This analysis was performed twice according to two different levels of successful response: undetectable plasma HIV viremia (Panel A); and HIV RNA that is detectable at <50 copies/mL (Panel B). Since survival analysis considers the time interval and failure rate over time, only data from patients with at least two consecutive observations could be used for this analysis. All patients’ data were used for the rest of the inferential analysis.

The relationship between the HIV viremia class and the CD4+ cell count, as outcome variables and other candidate predictor variables (patient cohort, hepatitis B core and hepatitis C serology, gender, and CD4+ nadir), was investigated using multivariate analysis of variance (MANOVA), nested according to the follow-up study point (T6, T12, T18, T24). The MANOVA results were used to identify potential candidate predictors in successive regression analysis.

The relationship between the CD4+ cell count and candidate predictor variables (patient cohort, hepatitis B core serology, and CD4+ nadir) was investigated using multilevel linear regression, nested according to the follow-up study point (T6, T12, T18, T24).

Lastly, the relationship between HIV viremia class and other candidate predictor variables (nadir and patient’s cohort) was investigated using a hierarchical ordered logistic model, nested according to the follow-up study point (T6, T12, T18, T24).

Statistical analyses were performed using Stata v. 17.0. (StataCorp., College Station, TX, USA; https://www.stata.com; 2021) and MedCalc^®^ Statistical Software version 22.003 (MedCalc Software Ltd., Ostend, Belgium; https://www.medcalc.org; accessed on 16 May 2023).

## 3. Results

A total of 329 PLWH had plasma HIV RNA that was undetectable or <50 copies/mL at T0: 98 were excluded because of lack of virological data of the 12 months before the switch; 32 patients had positive HBsAg; and 2 had positive HCV RNA at the switch. A total of 197 patients were included in the study, and they were immunocompetent (median CD4+ cell count was 677 cells/mm^3^). HIV RNA at T0 was undetectable in 108 patients. Most of the 197 patients (122, 61.9%) were on a previous INSTI-based regimen (Table 1).

One reason for switching was reported for 158 PLWH (80.2%), two reasons for twenty-five PLWH (12.7%), three reasons for one PLWH (0.5%), and no data were available for thirteen PLWH (6.6%). Overall, treatment optimization (69, 35%) and simplification to a single-tablet regimen (62, 31.5%) were the most frequently reported reasons (Table S1).

### 3.1. Plasma HIV RNA Control in the 12 Months before the Switch, and Virological Response to BIC/FTC/TAF

At T0, 108 patients had undetectable HIV RNA and 89 had HIV RNA < 50 copies/mL.

In the previous 12 months, 67 (34%) patients had always undetectable HIV RNA, 99 (50.3%) had at least one test showing <50 copies/mL, and 31 (15.7%) had at least one test showing ≥ 50 copies/mL. The median number of viral loads per patient in the 12 months prior to switch was three (IQR 2–3). Plasma HIV RNA undetectability was more frequent than plasma HIV RNA < 50 copies at T0 in PLWH with always-undetectable HIV RNA in the 12 months before the switch (45.4% vs. 20.2%, *p* = 0.0002). Conversely, plasma HIV RNA < 50 copies at T0 was more frequent in PLWH with at least one test < 50 copies/mL HIV RNA in the 12 months before the switch (64% vs. 38.9%, *p* = 0.0005) (Table 2).

Looking at the follow-up after the switch, plasma HIV RNA undetectability was most frequently detected in patients with undetectable HIV RNA at T0 with respect to patients with HIV RNA < 50 copies/mL at T0. At T6, T12, and T18, the differences were statistically significant (*p* < 0.0001, *p* = 0.0088, and *p* = 0.0327, respectively). Analysis via plasma HIV RNA control in the 12 months before the switch confirmed the higher frequency of plasma HIV RNA undetectability in patients with this optimal virologic response (Table 3 and Table 4).

Six (3%) patients stopped BIC/FTC/TAF due to the treating physician’s decision. A detailed description is reported in Table 5.

A few patients (9, 4.6%) had a plasma HIV RNA test showing > 100 copies/mL at follow-up. A value > 1000 copies/mL was detected in 3 PLWH, which was, in all cases, related to voluntary treatment suspension (Table 6).

### 3.2. Plasma HIV RNA Control in the 12 Months before the Switch and Virological Response to BIC/FTC/TAF

**Figure 1:**
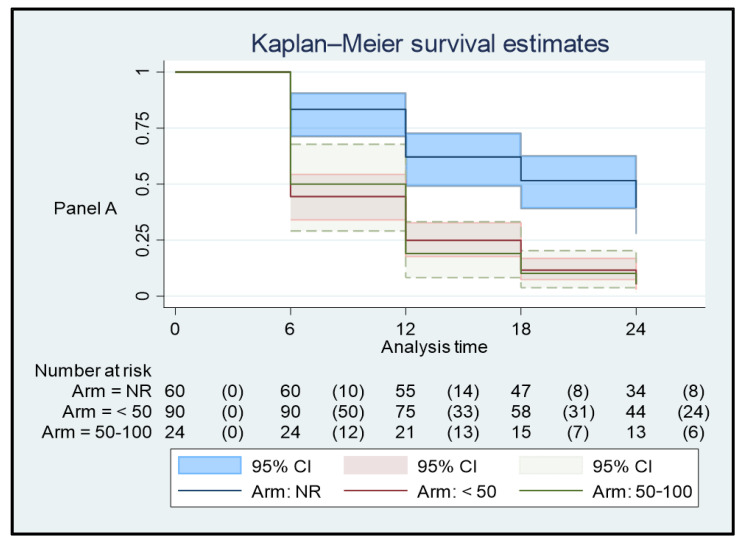

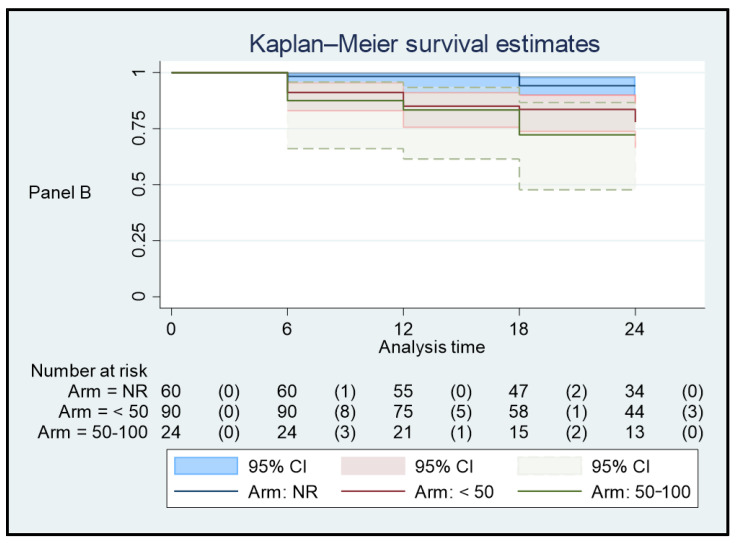
Kaplan–Meier survival estimates of virologic response during the 24 months of follow-up according to two different endpoints defining a successful response: plasma HIV viremia undetectable (Panel A); and HIV RNA detectable < 50 copies/mL (Panel B).

An analysis of the HIV RNA value after the switch was performed by applying two different endpoints as successful responses at T0, T6, T12, T18, and T24: undetectable and detectable HIV RNA at <50 copies/mL. The first approach showed a significantly higher frequency of HIV RNA undetectability throughout the 24 months of follow-up in patients with all test with undetectable HIV RNA (*p* < 0.001), with respect to the patients with plas-ma detectable HIV RNA before switch. The incidence rate ratio was 2.07; no difference was found between the patient groups when a higher viremia threshold was chosen. These data are detailed in Table 7 and Figure 1.

**Figure 2:**
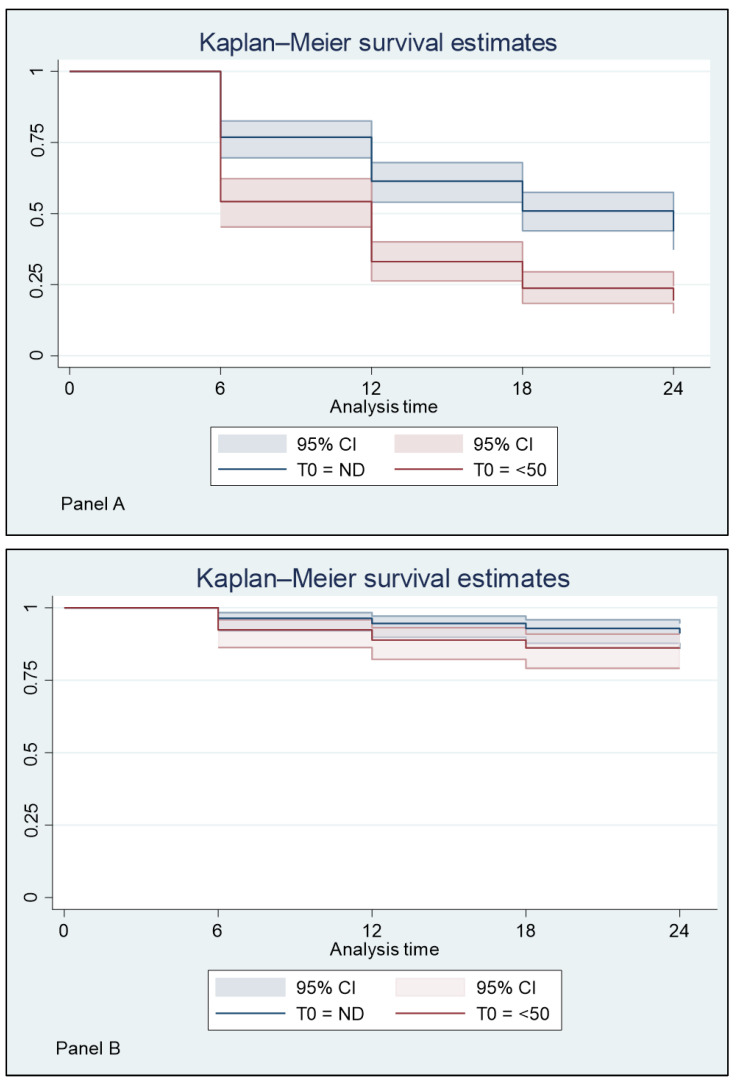
Kaplan–Meier survival estimates of virologic response during the 24 months of follow-up, according to two different endpoints defining a successful response: undetectable plasma HIV viremia (Panel **A**); and HIV RNA detectable at <50 copies/mL (Panel **B**) by the T0 viral load.

The analysis performed evaluating outcomes by the T0 viral load confirmed data described above and elaborated by the previous 12 months before T0 (Table 8 and Figure 2).

HBcAb (positivity vs. negativity), HCV serology (positivity vs. negativity), the absolute number of CD4+ cells, and HIV RNA at T0 (ND vs. HIV RNA < 50 copies/mL) were the four variables evaluated as predictors of the outcomes mean CD4+ cell count value and HIV RNA control (defined as undetectability vs de-tectability) at T6, T12, T18, and T24. The MANOVA results suggested that HIV RNA and the nadir CD4 cell count could be candidate predictors for both outcomes at the same time. A linear hierarchical model describing CD4+ cell count and viremia in relation to the candidate predictor variables is reported in Table 9, and CD4+ cell counts are reported in Table 10. When plasma HIV RNA control variable was plasma HIV RNA at T0 nadir cell count confirmed its role in predict-ing undetectable plasma HIV RNA during follow up (*p* < 0.001).

**Figure 3:**
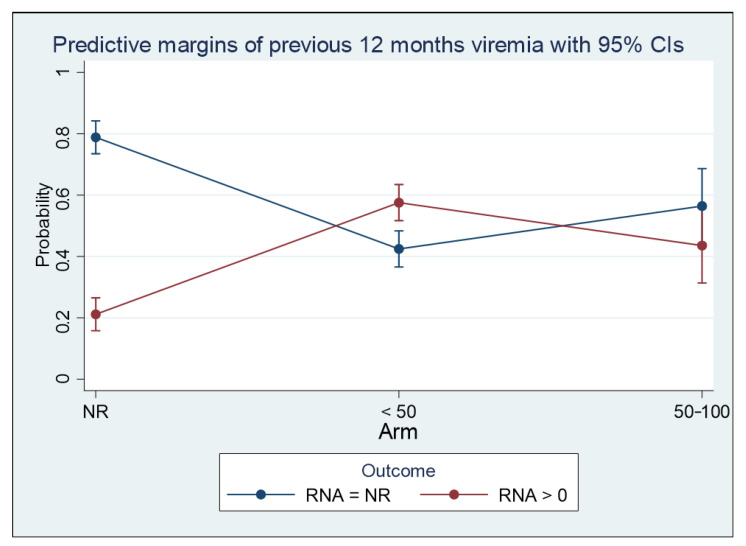
Marginal effect of plasma HIV viremia control in the 12 months before the switch on plasma HIV RNA control at T6, T12, T18, and T24. Outcome is defined as undetectable HIV RNA (Panel A).

Undetectability in the 12 months before the switch was associated with an 80% probability of maintaining undetectability; however, patients with detectable HIV RNA had a 50% possibility of achieving HIV RNA undetectability in at least one of the follow-up study points (Figure 3).

**Figure 4:**
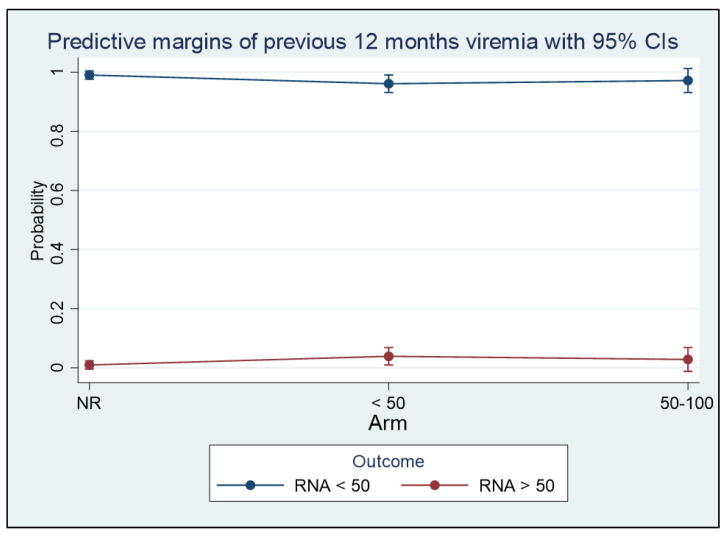
Description of the marginal effect of plasma HIV viremia control in the 12 months before the switch on the plasma HIV RNA control at T6, T12, T18, and T24, defined as HIV RNA detectable at < 50 copies/mL (Panel B).

When the evaluated outcome was HIV RNA < 50 copies/mL, we observed that almost all PLWH with always-undetectable plasma HIV RNA in the 12 months before the switch achieved this goal, and the probability was >90% whichever the virologic control in the 12 months before (Figure 4).

The roles of HBcAb status and HCV serology were also analyzed using a classical survival analysis approach according to the different levels of plasma HIV viremia control, and no influence was found on either outcome (Table 11).

### 3.3. Influence of ART before the Switch to BIC/FTC/TAF

The influence of ongoing ART before the switch was evaluated in the 167 PLWH treated with 2NRTI+INSTI (n = 100), 2 NRTI+NNRTI (n = 37), and 2 NRTI+PI (n = 30) before the switch. ART INSTI-based treatment was the most common ongoing regimen before the switch, except in PLWH who had always undetectable plasma HIV viremia the year prior and plasma HIV RNA < 50 cop-ies/ml at T0 (subgroup previously ND) (Table S2).

Furthermore, we analyzed the frequency of HIV RNA undetectability according to ongoing ART before the switch in the 60 PLWH, whose all follow-up data were available.

Forty patients were treated with an INSTI-based regimen; regardless of HIV RNA at T0, 16 (40%) PLWH maintained undetectable HIV RNA. Notably, 12 of the 16 patients were also undetectable at T0.

Of twenty patients treated with a drug combination, not including INSTI, only two (10%) PLWH, both negative at T0, were always ND, a frequency significantly lower than that in patients treated with INSTI (*p* = 0.018918).

## 4. Discussion

In high-income countries such as Italy, almost all PLWH on ART achieve and maintain virological suppression. For this reason, we designed this study to separately analyze patients with undetectable plasma HIV viremia before switching to BIC/FTC/TAF. Herein, we discussed three aspects of the successful response to this regimen.

First, this study demonstrated that long-term always-undetectable plasma HIV RNA before the switch was the main factor predicting the detection of this virological figure during the 24-month follow-up period; starting treatment with BIC/FTC/TAF in place of the previous ongoing successful ART regimen was demonstrated to maintain the previously achieved complete viral suppression; therefore, every PLWH can be safely treated with the regimen considered as the best option on the basis of their own specific clinical characteristics in a real-world setting. Our data on the efficacy of BIC/FTC/TAF in maintaining pre-existing virological suppression in treatment-experienced PLWH are in accordance with those of Micán et al. [25], who showed that having plasma HIV RNA < 50 copies/mL before the switch was associated with the detection of this same HIV RNA value after 48 weeks of treatment.

The median age of patients included in our study was 53 years at the switch, but only 34 years at diagnosis. Moreover, recently, it has been observed that a significant proportion of the newly diagnosed PLWH are young [26,27,28,29]. Due to long life expectancy, there may be a curative therapy for HIV infection available in the future, but HIV reservoirs are a major obstacle to the eradication of HIV infection [30], and it has been described that PLWH with sustained undetectable viremia have lower total HIV-DNA levels [31]. We are aware that the definition of undetectable plasma HIV viremia which we adopted is not definitively correct because of the probable persistence of very low viral replication that is identifiable with ultrasensitive assays [32,33,34], but these methods are not routinely applied in clinical practice, and the undetectable level is the most stringent level among those adopted in clinical studies.

Interestingly, at T24, undetectable plasma HIV RNA frequency was comparable in PLWH with plasma HIV RNA < 50 copies/mL and with undetectable HIV RNA at T0. Although it is important to interpret these findings in the context of the study’s small size, this result suggests that the high forgiveness of the drug combination [35] and/or a bictegravir human plasma unbound fraction (able to cross cell membranes and act on pharmacological sanctuaries), which can reach 6.7% [36], may have an impact on the long-term virological response.

The second result of our work is that a higher CD4+ count at the nadir predicted the achievement of undetectable HIV RNA; the median nadir value in PLWH enrolled in our study was 235 cells/mm^3^, a value compatible with a low likelihood of immune activation [37], and this may be a possible explanation for these data, being some aspect of immune activation associated with residual viremia [38,39]. Armenia et al. [9] found no influence of the nadir on the virological response in a study on PLWH who were switched to BIC/FTC/TAF; however, their statistical approach was different from ours because the nadir was evaluated as a dichotomic variable (≤200 cells/mm^3^ vs. >200 cells/mm^3^) and the authors evaluated its role in virological rebound (2 consecutive HIV RNA > 50 copies/mL or one test > 200 copies/mL); conversely, we considered CD4+ cell count as a continuous variable, and we considered the achievement of HIV RNA undetectability as an outcome during the 24-month follow-up period.

Finally, we observed that HBcAb positivity did not influence the virological response, a different result from that previously reported by our co-authors—Malagnino et al. [16,39]—in PLWH switching to a two-drug ART regimen containing lamivudine. These authors reported that HBcAb-positive PLWH had an OR of 2.7 after a 24-month follow-up period [16], and of 7.46 after 48 months [39] of HIV RNA detectability with respect to HBcAb-negative PLWH. A possible explanation for the lack of a role of previous HBV infection in our study is the presence of two drugs, TAF and FTC (both with antiviral activity against HBV), in the regimen started after the switch with respect to the treatment with lamivudine alone. HBcAb-positive PLWH could be patients with occult HBV infection and, therefore, with intermittent and very low HBV-DNA (generally < 200 IU/mL) [40]. Prolonged lamivudine therapy is associated with a risk of escape mutant emergence [41,42]. An alternative hypothesis for the different influences of HBcAb positivity on HIV viremia could be the HIV reservoir load in hepatocytes; Zerbato et al. [43] demonstrated the presence of HIV DNA in 61% of PLWH after more than 3 years of ART and virological response (plasma HIV RNA <50 copies/mL). In any case, treatment with BIC/FTC/TAF is safe in PLWH with positive HBcAb, and this result is important in light of the possible permanent or intermittent loss of HBcAb occurring in these patients [44].

Low-level viremia and blip are two laboratory results which are important in predicting virologic failure in patients on ART. This is an object of debate because of contrasting results which have been published, possibly due to the heterogeneity of the study designs and to the involvement of unknown factors associated with the suboptimal control of viral replication [45,46,47]. Here, we discuss data from recent studies focusing on the influence of uncontrolled HIV viremia as blip (isolated plasma HIV RNA value < 1000 copies/mL with a return to undetectable levels) or as low-level viremia (plasma HIV RNA value 50–200 copies/mL) as defined by IAS-USA [13].

A recent retrospective analysis [48] analyzing a European cohort of 22,523 patients starting ART, and with 81,837 person-years of follow-up (2.8 years as median), described low-level viremia in 7% and blips in 16% of patients. An increased risk of virological failure (defined as two consecutive plasma HIV RNA values ≥ 200 copies/mL or a single HIV RNA value ≥ 1000 copies/mL) was reported in association with both categories of uncontrolled viremia. A slightly different definition of virologic failure (two consecutive viral loads > 500 copies/mL) was applied by Fleming et al. [49] in a study enrolling 2795 patients (8423 person-years of outpatient care) attending clinics in the United States: 5.4% of patients experienced LLV; 19.9% patients experienced blips to 51–200 copies/mL; and 118 (4.2%) patients experienced blips to 201–500 copies/mL. Overall, LLV was associated with virologic failure, but blips were not; however, the association was significant in the cohort of patients with blips ranging from 201 to 500 copies/mL and no LLV. A third study including 10.124 Chinese patients with a median follow-up duration > 4 years and a median interval of 6–12 months between samples collection reported a higher frequency of virologic failure [50]. According to the authors, statistical significance could not be reached because of the small sample size (mean LLV rate was 6.2%). Conversely, another study set in China on 2155 patients (median follow-up time of 3.4 years) showed no increase in virological failure in patients with LLV (which has a frequency of 20.2%) or with blip. Notably, virological failure was defined as two or more consecutive plasma HIV RNA values > 1000 copies/mL [51]. Chen et al. [52] also confirmed this lack of association in a cohort of 1485 patients in Taiwan: the study period lasted 18 months, and 1.4% of the 3646 HIV RNA tests performed met the criteria of LLV. The correlation between blips and LLV was analyzed by Dijkstra et al. [53] in an observational cohort study which enrolled 1661 patients attending a Dutch hospital: 308 blips were described in 247 patients during 3405 treatment courses with a mean follow-up of 2.6 years. Interestingly, these authors showed that blip was not associated with a higher frequency of consecutive plasma HIV RNA values > 50 copies/mL (not classified as virologic failure) or virologic failure within that same treatment course.

Taken together, these data suggest that a percentage of patients did not achieve a successful virological response despite a presumed adherence to treatment. A definite role is difficult to determine, but low-level viremia and blip seem to have different clinical significance, and quantification may be important for the latter. In any case, the interplay between blip and LLV seems multifaceted, and may possibly be different even within a single patient.

The limitations of our study include the retrospective design of the analysis and the exclusion of patients lacking plasma HIV viremia control data reported in sanitary records in the 12 months before the switch. A portion of this period and of the study period itself occurred during the COVID-19 pandemic, and outpatients controls could not be performed regularly and or/reported in clinical charts. On the other hand, we included in the study only PLWH who had previous virologic monitoring results available with the aim to definitely select a cohort with an optimal response to treatment and adherence.

## 5. Conclusions

The predictive role of undetectable HIV viremia in the year before the switch in long-term virological success suggests that the switch could be programmed and possibly delayed when simplification is the reason for and on a case by case basis, in accord with the patient to achieve persistent plasma HIV RNA undetectability. Moreover, undiagnosed loss of HBcAb has no detrimental consequences on the response to BIC/FTC/TAF. A longer follow-up period is certainly needed in order to confirm the predictive role of the factors studied.

## Figures and Tables

**Table 1:** Main characteristics of the study population (n = 197).

Characteristics	n (%)
Male, n (%)	140 (71.1)
Caucasian origin, n (%)	162 (82.2)
HBsAg negative and HBcAb positive *, n (%)	58 (33)
Positive HCV serology, n (%)	29 (14.7)
Age at HIV diagnosis (years), median (IQR)	34 (26–43)
Age at switching (years), median (IQR)	53 (41–59)
Absolute number of CD4+ T cell count at nadir (cells/mm^3^), median (IQR)	235 (73–346)
CD4+ T cell count (cells/mm^3^) at switch, median (IQR)	677 (490–880)
Pre-switch therapy: 2 NRTI+INSTI	100 (50.8)
Pre-switch therapy: 2 NRTI+NNRTI	37 (18.7)
Pre-switch therapy: 2 NRTI+PI	30 (15.2)
Pre-switch therapy: other regimen including INSTI	22 (11.2)
Pre-switch therapy: other regimen not including INSTI	8 (4.1)

NRTI: nucleotide reverse transcriptase inhibitors; INSTI: including integrase strand transfer inhibitors; NNRTI: nonnucleoside reverse transcriptase inhibitors; PI: protease inhibitors. * Data available for 176 patients.

**Table 2:** Description of the frequency of the three different virologic patterns in the 12 months before the switch in patients with undetectable plasma HIV RNA and those with detectable plasma HIV RNA at <50 copies/mL at T0. Data are expressed as absolute numbers and percentages.

Virologic Control at T0	Always Undetectable HIV RNA in the 12 Months before Switch (n = 67)	At Least One Test < 50 Copies/mL in the 12 Months before Switch (n = 99)	At Least One Test ≥ 50 Copies/mL in the 12 Months before Switch (n = 31)
Undetectable HIV RNA at T0, n = 108	49 (45.4)	42 (38.9)	17 (15.7)
Detectable HIV RNA < 50 copies/mL at T0, n = 89	18 (20.2)	57 (64)	14 (15.8)

**Table 3:** Description of virological responses at T6, T12, T18, and T24. Data were reported independently for each follow-up time and classified according to plasma HIV RNA at T0 (not detectable or detectable at < 50 copies/mL).

	T6 (158 Patients)				
Plasma HIV RNA Control at T0	ND	<50 Copies/mL	50–100 Copies/mL	101–1000 Copies/mL	>1000 Copies/mL
HIV RNA NR at T0 (n = 87)	61 (70.1)	21 (24.1)	2 (2.3)	3 (3.5)	0
HIV RNA < 50 copies/mL at T0 (n = 71)	25 (35.2)	39 (54.9)	5 (7.1)	2 (2.8)	0
	T12 (143 patients)				
Plasma HIV RNA control at T0	ND	<50 copies/mL	50–100 copies/mL	101–1000 copies/mL	>1000 copies/mL
HIV RNA NR at T0 (n = 78)	53 (68)	23 (29.5)	2 (2.5)	0	0
HIV RNA < 50 copies/mL at T0 (n = 65)	30 (46.2)	31 (47.7)	2 (3.1)	1 (1.5)	1 (1.5)
	T18 (115 patients)				
Plasma HIV RNA control at T0	ND	<50 copies/mL	50–100 copies/mL	101–1000 copies/mL	>1000 copies/mL
HIV RNA NR at T0 (n = 64)	44 (68.8)	17 (26.6)	2 (3.1)	0	1(1.6)
HIV RNA < 50 copies/mL at T0 (n = 51)	25 (49)	24 (47)	1(2)	0	1(2)
	T 24 (91 patients)				
Plasma HIV RNA control at T0	ND	<50 copies/mL	50–100 copies/mL	101–1000 copies/mL	>1000 copies/mL
HIV RNA NR at T0 (n = 54)	34 (63)	17 (31.5)	2 (3.7)	1 (1.8)	0
HIV RNA < 50 copies/mL at T0 (n = 37)	19 (51.4)	18 (48.6)	0	0	0

**Table 4:** Description of virological response at T6, T12, T18, and T24 according to plasma HIV RNA control in the 12 months before the switch classified as follows: not detected (undetectable HIV RNA in all the tests); plasma HIV RNA < 50 copies/mL (at least one test with a value < 50 copies/mL); and plasma HIV viremia ≥ 50 copies/mL (at least one test with a value ≥ 50 copies/mL). Data are reported independently for each follow-up time.

	T6 (158 Patients)				
Plasma HIV RNA Control in the 12 Months before Switch	ND	<50 Copies/mL	50–100 Copies/mL	101–1000 Copies/mL	>1000 Copies/mL
HIV RNA ND (n = 56)	46 (82.1)	9 (16.1)	0	1 (1.8)	0
HIV RNA < 50 copies/mL (n = 81)	31 (38.3)	42 (51.9)	4 (4.9)	4 (4.9)	0
HIV RNA ≥ 50 copies/mL (n = 21)	9 (42.9)	9 (42.9)	3 (14.2)	0	0
	T12 (143 patients)				
Plasma HIV RNA control in the 12 months before switch	ND	<50 copies/mL	50–100 copies/mL	101–1000 copies/mL	>1000 copies/mL
HIV RNA ND (n = 52)	38 (73.1)	14 (26.9)	0	0	0
HIV RNA < 50 copies/mL (n = 71)	38 (53.5)	28 (39.4)	4 (5.6)	1 (1.4)	0
HIV RNA ≥ 50 copies/mL (n = 20)	7 (35)	12 (60)	0	0	1 (5)
	T18 (115 patients)				
Plasma HIV RNA control in the 12 months before switch	ND	<50 copies/mL	50–100 copies/mL	101–1000 copies/mL	>1000 copies/mL
HIV RNA ND (n = 45)	37 (82.2)	6 (13.3)	1 (2.2)	0	1 (2.2)
HIV RNA < 50 copies/mL (n = 56)	25 (44.6)	30 (53.6)	0	0	1 (1.8)
HIV RNA ≥ 50 copies/mL (n = 14)	7 (50)	5 (25.7)	2 (14.3)	0	0
	T24 (91 patients)				
Plasma HIV RNA control in the 12 months before switch	ND	<50 copies/mL	50–100 copies/mL	101–1000 copies/mL	>1000 copies/mL
HIV RNA ND (n = 34)	26 (76.5)	8 (23.5)	0	0	0
HIV RNA < 50 copies/mL (n = 44)	20 (45.5)	21 (47.7)	2 (4.5)	1 (2.3)	0
HIV RNA ≥ 50 copies/mL (n = 13)	7 (53.8)	6 (46.2)	0	0	0

**Table 5:** Description of PLWH who stopped treatment with BIC/FTC/TAF.

PLWH	Caucasian	Last Follow-Up Point Available	Reason
M, 66 years	Yes	T6: HIV RNA ND	Stopped BIC/FTC/TAF (reason not reported), restarted previous treatment (RPV/TAF/FTC)
M, 55 years	Yes	T18: HIV RNA < 50 copies/mL	Stopped BIC/FTC/TAF due to side effects, started DTG/3TC
M, 52 years	No	T12: HIV RNA ND	Stopped BIC/FTC/TAF due to side effects, started DTG/3TC
M, 44 years	Yes	T6: HIV-RNA 50–100 copies/mL	Stopped BIC/FTC/TAF due to side effects, started DOR, 3TC, TDF
M, 49 years	Yes	T6: HIV RNA 101–1000 copies/mL	Stopped BIC/FTC/TAF due to therapeutic failure, started DOR, 3TC, TDF
F, 58 years	Yes	T6: HIV RNA ND	Stopped BIC/FTC/TAF due to side effect (weight increase), restarted TAF/FTC/RAL

RPV: rilpivirine; DTG: dolutegravir; 3TC: lamivudine; DOR: doravirin; TDF: tenofovir disoproxil fumarate; RAL: raltegravir.

**Table 6:** Time course of PLWH who had a first plasma HIV RNA value ranging from 101 copies/mL. to more than 1000 copies/mL.

PLWH	Caucasian	First Detection of HIV-RNA > 101 Copies/mL	Reason	Follow-Up
M, 63 years	Yes	T6: 101–1000 copies/mL	Not adherent to therapy	No further data available
F, 33 years	Yes	T6: 101–1000 copies/mL	Blip ^a^	T12: HIV RNA < 50 copies/mL; T18: HIV RNA ND; T24:HIV RNA ND
M, 63 years	Yes	T6: 101–1000 copies/mL	Blip ^a^	T12 due on May 2023
M, 49 years	Yes	T6: 101–1000 copies/mL	Therapeutic failure	Started DOR, 3TC, TDF
F, 73 years	Yes	T6: 101–1000 copies/mL	Possible low adherence	T12: HIV RNA 101–1000 copies/mL, T18:HIV RNA < 50 copies/mL, T24 due on April 2023
F, 44 years	Yes	T12: >1000 copies/mL	Treatment stopped due to patient’s decision	Not available
M, 55 years	Yes	T18: >1000 copies/mL	Treatment stopped due to patient’s decision and then restarted	T24: HIV RNA < 50 copies/mL
M, 27 years	Yes	T18: >1000 copies/mL	Treatment stopped due to patient’s decision and then restarted	T24: HIV RNA ND
M, 35 years	Yes	T24: 101–1000 copies/mL	Blip ^a^	Not applicable

ND: not detectable. ^a^ Blip is a single plasma HIV RNA value between 50 and 500 copies/mL followed by a viral load not detectable. Sanitary records reported a plasma HIV RNA undetectable in a test performed after a short time.

**Table 7:** Survival analysis with multiple-failure-per-subject design. Two different endpoints were applied as successful responses: undetectable HIV viremia (Panel A); and plasma HIV RNA detectable at <50 copies/mL (Panel B).

**(** **Panel A)**			
**Plasma HIV RNA in the 12 Months before the Switch**	**Time at Risk**	**Rate**	**Patients**
All tests with undetectable plasma HIV RNA	1.260	0.0301587	67
At least one test with plasma HIV RNA < 50 copies/mL	1.722	0.0720093	99
At least one test with plasma HIV RNA ≥ 50 copies/mL	486	0.0720165	31
Total	3.468	0.0568051	197
**(Panel B)**			
**Plasma HIV RNA in the 12 Months before the Switch**	**Time at risk**	**Rate**	**Patients**
All tests with undetectable plasma HIV RNA	1.260	0.0015873	67
At least one test with plasma HIV RNA < 50 copies/mL	1.722	0.0087108	99
At least one test with plasma HIV RNA ≥ 50 copies/mL	486	0.0102881	31
Total	3.468	0.0063437	197

**Table 8:** Survival analysis with multiple-failure-per-subject design. Two different endpoint as successful responses were applied: undetectable HIV viremia (Panel A); and plasma HIV RNA detectable < 50 copies/mL (Panel B).

**(** **Panel A)**			
**Plasma HIV RNA at T0**	**Time at Risk**	**Rate**	**Patients**
Plasma HIV RNA undetectable	2592	0.35	108
Plasma HIV RNA < 50 copies/mL	2136	0.59	89
Total	4728	0.46	197
**(Panel B)**			
**Plasma HIV RNA at T0**	**Time at risk**	**Rate**	**Patients**
Plasma HIV RNA undetectable	2592	<0.01	108
Plasma HIV RNA < 50 copies/mL	2136	<0.01	89
Total	4728	<0.01	197

**Table 9:** Linear hierarchical model describing the influence of HbcAb status, HCV and/or HBV serology status, CD4+ cell count at nadir, and plasma HIV viremia control in the 12 months before the switch to BIC/FTC/TAF.

*Predictor Variables*	*Coefficient*	*95% CI*	*p-Value*
***Viremia in previous 12 months (ref.: always ND)***	-	-	-
*<50 copies/mL*	123.25	67.29–179.20	**<0.001**
*50–100 copies/mL*	101.46	12.96–189.96	**0.025**
***Nadir cell count***	1.06	0.92–1.20	**<0.001**
***HCV status (ref.: negative)***	-	-	-
*positive*	−62.90	−154.85–29.04	0.18
***HBV status (ref.: negative)***	-	-	-

**Table 10:** Description of mean CD4+ cell count at T0 and the follow-up study points (T6, T12, T18, and T24) according to plasma HIV RNA control in the 12 months before T0.

Viremia in Previous 12 Months	T0			T6			T12			T18			T24			
	Mean	SE	Δ	Mean	SE	Δ	Mean	SE	Δ	Mean	SE	Δ	Mean	SE	Δ	Δ (T24-T0)
HIV RNA ND	577	54	-	657	63	80	672	75	14	719	85	46	705	75	−14	127
At least one plasma HIV RNA < 50 copies/mL	764	90	-	804	85	40	778	83	−26	809	90	30	826	95	17	62
At least one plasma HIV RNA ≥ 50 copies/mL	822	103	-	792	86	−30	843	121	51	867	120	24	828	101	−39	5

**Table 11:** Description of Mantel–Haenszel estimates of the rate ratio and the degree of association between the different levels of plasma HIV viremia in the 12 months before the switch and HBcAb status and HCV serology.

*Viremia in Previous 12 Months*	*Time at Risk*	*Incidence Rate*	*N*
*ND*	1260	0.030	67
*<50 copies/mL*	1722	0.072	99
*≥50 copies/mL*	486	0.072	31
***Control variables***	*Rate Ratio*	*95% CI*	*p-value*
*HBV positive*	0.9	0.6–1.2	0.254
*HCV positive*	1.2	0.8–1.8	0.525
*Male gender*	1.2	0.9–1.6	0.568

## Data Availability

The raw data on demographics and clinical status of participants are protected and not available due to data privacy laws. The processed data are available from the corresponding author upon reasonable request.

## Supplementary Materials

The following supporting information can be downloaded at: https://www.mdpi.com/article/10.3390/v15081727/s1, Table S1: Description of the reason for switching according to the frequency; Table S2: Description the different distribution of two patterns of plasma HIV RNA control in the 12 months before switch and at T0: subgroup ND (ND in the 12 months before and ND at T0), subgroup previously ND (ND in the 12 months before and detectable at T0), subgroup previously detectable (any HIV RNA positive detection in the 12 months before and ND at T0), always detectable (any HIV RNA positive detection in the 12 months before and HIV RNA < 50 copies/mL at T0).
