# Supplementary material for: Predicting Factors of Plasma HIV RNA Undetectability after Switching to Co-Formulated Bictegravir, Emtricitabine, and Tenofovir Alafenamide in Experienced HIV-1 Patients: A Multicenter Study
Source: Viruses. 2023 Aug 12;15(8):1727. doi: 10.3390/v15081727 (PMC10458950; doi:10.3390/v15081727)
Supplement: Supplementary file 1 [file viruses-15-01727-s001.zip › viruses-2523762-supplementary.pdf]

Table S1. Description of the reason for switching according to the frequency.

One reason (n=158)		
	N	%
Treatment optimization	60	38
Simplification to Single Tablet Regimen	53	33.5
Side effects	15	9.5
Risk of virologic failure	11	7
Prevention of cardiovascular risk	8	5
Drug-Drug interactions	5	3.2
Drug unavailability	3	1.9
Patient preference	3	1.9
Two reasons (n=25)		
Simplification to Single Tablet Regimen and prevention of cardiovascular risk	6	24
Side effects and prevention of cardiovascular risk	5	20
Treatment optimization and risk of virological failure	3	12
Treatment optimization and prevention of cardiovascular risk	3	12
Simplification to Single Tablet Regimen and side effects	3	12
Side effects and risk of virological failure	2	8
Treatment optimization and drug-drug interactions	1	4
Treatment optimization and side effects	1	4
Drug-Drug interactions and prevention of cardiovascular risk	1	4
Three reasons (n=1)		
Treatment optimization, side effects and prevention of cardiovascular risk	1	100

Table S2. Description the different distribution of two patterns of plasma HIV RNA control in the 12 months before switch and at T0: subgroup ND (ND in the 12 months before and ND at T0), subgroup previously ND (ND in the 12 months before and detectable at T0), subgroup previously detectable (any HIV RNA positive detection in the 12 months before and ND at T0), always detectable (any HIV RNA positive detection in the 12 months before and HIV RNA < 50 copies/ml at T0).

	2NRTI+INSTI (n=100)	ART not ISTI based (n=67)	*2NRTI+NNRTI (n=37)*	*2NRTI+PI (n=30)*
Subgroup ND (42 PLWH), n (%)	24 (57.2)	18 (42.8)	*14 (33.3)*	*4 (9.5)*
Subgroup previously ND (16 PLWH), n (%)	7 (43.8)	9 (56.2)	*5 (31.2)*	*4 (25)*
Subgroup previously detectable (49 PLWH), n (%)	35 (71.4)	14 (28.6)	*3 (6.1)*	*11 (22.4)*
Subgroup always detectable (60 PLWH), n (%)	34 (56.7)	26 (43.3)	*15 (25)*	*11 (18.3)*
